# Newly Developed Techniques on Polycondensation, Ring-Opening Polymerization and Polymer Modification: Focus on Poly(Lactic Acid)

**DOI:** 10.3390/ma9030133

**Published:** 2016-02-26

**Authors:** Yunzi Hu, Walid A. Daoud, Kevin Ka Leung Cheuk, Carol Sze Ki Lin

**Affiliations:** 1 School of Energy and Environment, City University of Hong Kong, Tat Chee Avenue, Kowloon, Hong Kong, China; yunzihu2-c@my.cityu.edu.hk (Y.H.); wdaoud@cityu.edu.hk (W.A.D.); 2 The Institute of Textiles and Clothing, The Hong Kong Polytechnic University, Hung Hom, Kowloon, Hong Kong, China; kevinkevin.email@gmail.com

**Keywords:** polymer synthesis, polycondensation, ring-opening polymerization, modification technique, poly(lactic acid)

## Abstract

Polycondensation and ring-opening polymerization are two important polymer synthesis methods. Poly(lactic acid), the most typical biodegradable polymer, has been researched extensively from 1900s. It is of significant importance to have an up-to-date review on the recent improvement in techniques for biodegradable polymers. This review takes poly(lactic acid) as the example to present newly developed polymer synthesis techniques on polycondensation and ring-opening polymerization reported in the recent decade (2005–2015) on the basis of industrial technique modifications and advanced laboratory research. Different polymerization methods, including various solvents, heating programs, reaction apparatus and catalyst systems, are summarized and compared with the current industrial production situation. Newly developed modification techniques for polymer properties improvement are also discussed based on the case of poly(lactic acid).

## 1. Introduction

The enormous disposal of plastic trash, around 140 million tons per year [1], ends up as solid waste disposal on land and ocean dumping which leads to long-term contamination in soils and water environment. Therefore, in recent decades, biodegradable plastics (BDPs) derived from renewable sources have undergone extensive investigations in academic research and industry. Instead of disposal in landfills or by incineration, biodegradable plastic could be decomposed by bacteria or other living microorganisms due to their potentially hydrolysable ester bonds [2].

Among numerous bioplastics, poly(lactic acid) (PLA) is considered the most promising and popular material because of its ideal properties in use: low weight, low processing temperature (compared to metal and glass), no environmental pollution, good printability, and ease of conversion into different forms [3]. It has been produced in large scale by firms, such as Cargill Dow Polymers (Minnetonka, MN, USA), with wide applications from medical materials to disposable food tableware. However, at this stage, petroleum products still dominate the plastic market, due to the high production cost and limited properties of PLA and other BDPs [4].

Polymer synthesis known as polymerization is the process of connecting monomers into chain or network. Belonging to hydroxyl acids, the monomer of PLA is lactic acid (α-hydroxypropionic acid) with a hydroxyl acid at α position, facilitating its polymerization. PLA is usually synthesized by polycondensation and ring-opening polymerization. These two typical synthesis routes are also generally applied in other polymer production.

Therefore, this review takes PLA as the representative to evaluate the different synthesis methods newly developed in the period of 2005–2015. Four synthesis methods, direct polycondensation (DP), azeotropic polycondensation (AP), solid state polymerization (SSP) and ring-opening polymerization (ROP), will be mainly discussed to present the improved techniques and processes in polymer synthesis, especially for polyesters. Newly developed techniques in bulk modification and surface modification to enhance polymer properties are also presented in this review.

## 2. PLA Synthesis

**Figure 1:**
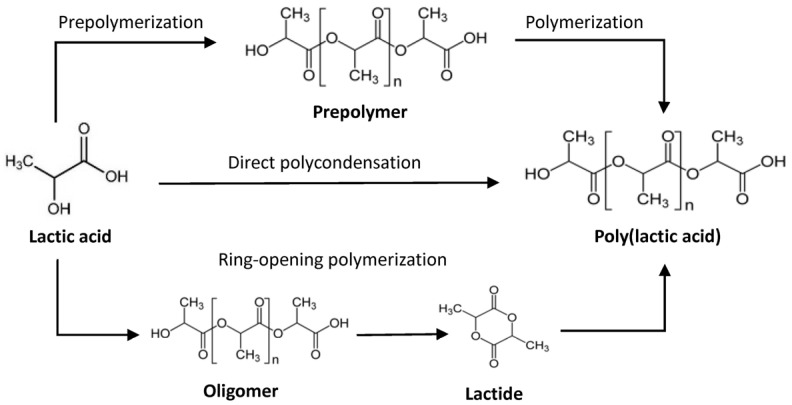
Routes of poly(lactic acid) (PLA) synthesis from lactic acid.

Generally, there are three routes to produce PLA polymers from lactic acid as shown in Figure 1. Direct condensation polymerization forms low molecular weight PLA. Two steps polymerization (e.g., SSP) can achieve higher molecular weight, but is still limited by the equilibrium reaction of polycondensation due to hydrolysis of ester bonds [5].

In 1932, a new method named ring-opening polymerization was firstly demonstrated by Carothers [6]. In this route, lactic acid is firstly polymerized to a low molecular weight oligomer, which is catalytically depolymerized through internal transesterification to lactide, in the “back-biting” reaction [7]. Then, the ring of lactide opens to form high molecular weight PLA. Actually, this is the most largely used route in PLA industrial production.

Although in the recent decade many studies have been progressively conducted in this area for technique improvement, there has been no summary or comparison of these synthesis methods. Therefore, this review aims strategically at filling the gap between laboratory research and industrial techniques.

## 3. Polymer Synthesis by Polycondensation

Polycondensation is polymer formation process by linking small molecules (monomers) together, accompanied by elimination of byproducts (e.g., water and alcohols). In case of PLA, polycondensation of lactic acid by connecting carboxyl and hydroxyl groups produces water byproduct simultaneously. Due to the difficulty in removing byproducts completely from the highly viscous reaction mixture, polymer produced through direct polycondensation is usually of low molecular weight (<50,000 g·mol^−1^) and low quality. In order to overcome this main disadvantage, numerous newly developed polycondensation methods have been proposed. In recent years, azeotropic polycondensation (AP) and solid state polymerization (SSP) are two main directions.

For AP approach, the water is removed efficiently by appropriate azeotropic solvents, by which the equilibrium between monomer and polymer is manipulated in organic solvent to produce polymer with relatively high molecular weight in one step. Besides, the temperature applied is allowed to be lower than polymer melting point, avoiding impurities caused by depolymerization and racemization [5]. Therefore, appropriate solvent is critical to performance conditions and polymer properties.

Normally, SSP consists of two steps: melt state to produce oligomer at high temperature (150–200 °C) and solid state to further increase molecular weight at temperature between the glass transition and melting point. In the second step, the prepolymer of relatively low molecular weight is semi-crystalline powder, chip, pellet or fiber, which is usually pulverized and thoroughly dried before heating. Hence, heat transfer and distribution among dry particles is highly efficient and homogeneous, resulting in high molecular weight [8]. Moreover, since the cyclisation, decomposition and other side reactions are limited at low temperature, SSP polymers usually have improved properties and purity.

### Recent Development in PLA Synthesis by Polycondensation

In the recent decade, polycondensation techniques have been improved with more efficient synthesis methods. Several modified methods have been reported to produce PLA with high molecular weight successfully. The selected methods starting from commercial lactic acid are summarized in Table 1.

Achmad *et al.* reported an improved direct polycondensation method producing PLA with molecular weight of 90,000 g·mol^−1^ even without any catalyst, initiator or solvent [9]. However, the cost of energy consumed for long time heating (>100 h at 200 °C) would be much higher than that of catalyst. With better heating effect, microwave-assisted synthesis was reported as a more efficient direct polycondensation method which resulted in PLA (*M*_w_ 16,000 g·mol^−1^) within 30 min [10]. This research also pointed out the enhanced catalytic effect of binary catalyst such as SnCl_2_/*p*-TsOH. Nevertheless, potential higher yield (>54%) and higher molecular weight needs further study.

In the AP approach, besides azeotropic solvents, a Soxhlet extractor with molecular sieve (3 Å) inside was also mounted simultaneously to remove trace water from refluxed solvent and over 30,000 g·mol^−1^ polymer was obtained [11]. Fukushima *et al.* pulverized prepolymer synthesized at melt-polycondensation stage to particles with diameter less than 150 µm and thoroughly dried them before SSP [8]. In SSP, the heating program started at 130 °C and rose to 160 °C stepwise as polymer melting point increasing, resulting PLA with *M*_w_ over 200,000 g·mol^−1^ [8], a breakthrough in polycondensation. Moreover, this team also suggested that a starting mixture of l-PLA and d-PLA in 1:1 ratio in solid state polycondensation could improve the polymer melting point from 160–170 °C to over 200 °C, indicating reinforced thermal stability [12]. These methods presented efficient improvements in the heating program and increasing the molecular weight by elimination of byproducts.

## 4. Polymer Synthesis by Ring-Opening Polymerization

Ring-opening polymerization is a propagation process of cyclic monomers initiated by different ions. As the reactive center of propagation, the terminal end of a polymer classifies the mechanism into anionic ROP, cationic ROP and radical ROP [13]. For PLA, the cyclic monomer is the intermediate namely lactide, which is the cyclic dimer of lactic acid. Particularly, the controlled ROP could lead to polymer with specific and desirable properties (e.g., refractive index, molecular weight) [14], making it very significant in polymer synthesis today. As mentioned above, for a high molecular weight PLA synthesis, the route through ROP of lactide is the most commonly used. Comparing with polycondensation, polymerization of lactide can produce polymer with wider range of molecular weight by controlling the purity of lactide and synthesis conditions, without chain coupling agent or azeotropic system. Therefore, ROP is applied by some PLA leading producers, such as Cargill Dow (Minnetonka, MN, USA) and Shimadzu (Kyoto, Japan) [15].

### 4.1. Recent Development in PLA Synthesis Method through Ring Opening Polymerization

PLA production through ROP has been developed for more than 80 years since it was firstly invented in 1935. The high molecular weight PLA was produced with improved purification method by DuPont in 1954 [16]. Since the reaction is sensitive to experimental conditions, including temperature, heating rate, inner pressure, catalyst and reagent, it is important to investigate the optimal manipulation. For this, various methods and catalysts have been studied. In 1992, Cargill (US) successfully applied ROP in industrial production and patented its method [17]. In 2005, Rajeev *et al.* summarized several typical methods of 1990s [4]. In his review, various catalyst, solvents and reaction temperature were summarized in relation to the molecular weight of PLA product. It revealed that using stannous octoate as a catalyst accomplished by heating at 150–210 °C could contribute to high molecular weight. In 2007, Gupta *et al.* summarized over one hundred catalysts used in lactide polymerization [5]. However, the given information was not elaborated enough to raise a comparison or advise the specific production process.

On the other hand, information regarding further study in particular production process and laboratory operating techniques is rarely reported in recent publications, due to the wide application of the traditional method (e.g., oil bath heating under vacuum environment with tin-based catalyst) in PLA industrial production. In order to fill the gap, recent publications and patents on newly developed ROP techniques are selected and summarized in this review.

#### 4.1.1. Lactide Synthesis

As the intermediate in ROP, lactide is of most significance in PLA production and its purity is critical to PLA properties. Therefore, synthesis of lactide and PLA is usually separated to purify the obtained lactide by recrystallization in proper solvent such as ethyl acetate and toluene. Accordingly, the processes of lactide and PLA production are summarized separately in Table 2 and Table 3. Based on the traditional method, the production and separation of lactide through depolymerization and distillation at high temperature (180–300 °C) under vacuum is quite time and energy consuming [5]. The newly developed laboratory techniques proposed several higher efficient or cost-effective production methods.

The first method in Table 2 is a Chinese patent, where catalysts used were chemically treated stones, such as acidified montmorillonite, which is a readily available lower cost material as compared to the typical tin-based catalyst. The second method was reported by researchers in Switzerland, in which a short path distillation is applied for lactic acid dehydration and reactive distillation of raw lactide, yielding 95%–97% (*w*/*w*), much higher than conventional condensation system [19]. However, higher amount of catalyst (3 wt %) and high temperature (250 °C) were still required. Hong *et al.* and Liang *et al.* used nitrogen gas flow to improve reaction efficiency by removing lactide from reaction system instantly [20,21]. Moreover, constant temperature heating device and modified microwave were also employed for better heating effect in recent years. The result revealed that both of these reaction setups could produce lactide at temperature lower than 210 °C [22,24]. Besides, microwave shortened the synthesis time by more than 50% [24]. Instead of using lactic acid as a starting material, Upare *et al.* investigated the production of lactide from alkyl lactate and the highest conversion yield of 46% was obtained from ethyl lactate, which is lower than the yield obtained using lactic acid as raw material (53%) [25].

#### 4.1.2. Polymerization of Lactide

All polymerization mentioned below started from purified and dried lactide (>99% purity). Initiators, such as solvents or trace amount of water, are required to work with catalyst to induce ROP process. Different types of initiators would lead to different reaction mechanisms, and they can be classified into three types: anionic polymerization, cationic polymerization and coordination–insertion mechanism [5]. The catalyst system mainly includes one or more components of metal powders, metal salts, Lewis acid, Lewis bases and organic compounds [16]. Contrary to polycondensation, molecular weight of PLA produced by ROP is not of positive correlation to long heating duration. Sanglard *et al.* found that PLA molecular weight did not always increase with time and the highest value, 172,663 g·mol^−1^, was obtained at 15 min under 170 °C [19]. Then the molecular weight decreased after 30 min and tended to be stable [19]. This indicated the polymerization of lactide could reach a plateau in short time and long heating duration may cause degradation instead.

**Figure 2:**
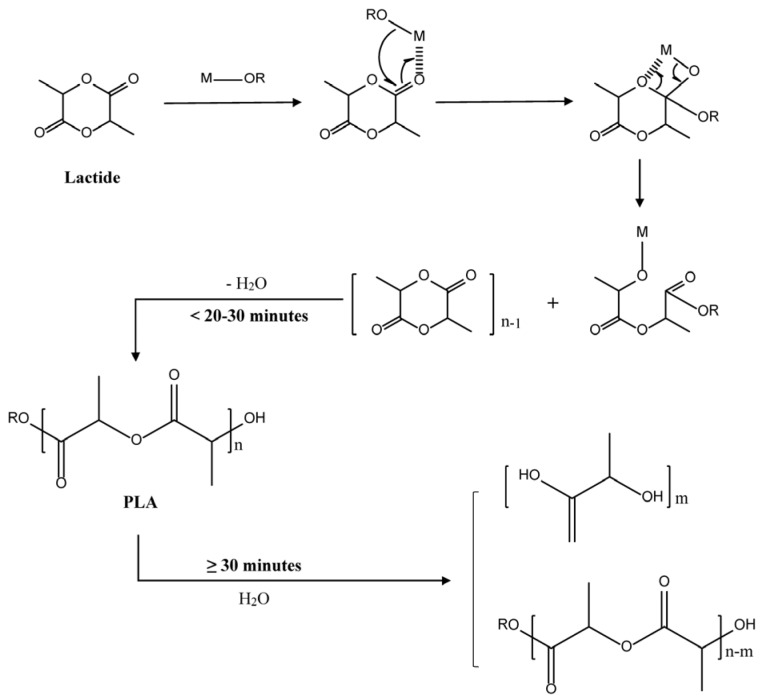
Proposed scheme of ROP for lactide.

As to the equipment, a twin-screwed extruder was used to conduct reactive extrusion which improved the conversion yield to 99% and shortened duration time to only 7 min, significantly improving the efficiency [26]. Korhonen *et al.* investigated polymerization with different co-initiators and proposed that more hydroxyl groups of co-initiators led to higher molecular weight (>400,000 g·mol^−1^) and faster polymerization (<5 h), without affecting the polymer thermal properties [27]. In contrast, Zhong *et al.* compared solution polymerization and solvent-free polymerization, from which higher molecular weight PLA was obtained with higher efficiency in solvent-free set [28]. Kaihara *et al.* successfully achieved PLA with high *M*_w_ (100,000 g·mol^−1^) only at 140 °C for 10 h, however, extremely low pressure (0.001 kPa) and dry condition requires longer period of preparation [29]. This study also pointed out lower temperature (≤140 °C) could contribute to improve molecular weight by avoiding decomposition resulted from back-biting reaction [29], whereas Korhonen *et al.* achieved better quality PLA (160,000 g·mol^−1^) at 200 °C for 1 h even without solvent as co-initiator [27]. In Korhonen’s research, the highest molecular weight was obtained at 15–20 min [27], the same trend as study result of Sanglard *et al.* [19]. That is possibly because higher temperature (170–200 °C) aggravates both chain-growth polymerization and decomposition, but polymerization has the priority at first. After 30 min, heat accumulation turns decomposition to dominate the reaction balance to decrease the polymer molecular weight. Figure 2 shows the process of this hypothesis (the arrows point out the dominating reaction), which requires further investigation for confirmation.

In terms of catalyst selection, apart from the mostly used organometallic catalyst (e.g., zinc and tin based), metal-free organocatalyst has undergone a renaissance in polymerization since 2005 [36]. Guanidine and amidine organocatalyst have proven highly effective towards ring-opening polymerization of cyclic esters, such as lactide [32]. According to research of Hedrick and Waymouth (2006), TBD enabled shorter ROP reaction time of 20 s to 1 min with conversion yield of 95%–99% [32]. Alcohol adducts of N-Heterocyclic carbenes (NHCs) also polymerized lactide into PLA within 10 min at room temperature [34]. The combined catalyst of bis-sulfonamide and tertiary amines could control the molecular weight of PLA under mild conditions [37]. Direct ROP of lactide with amine could prepare amine end-caped PLA and help to remove unreacted monomer without affecting polymer properties [38]. As a new class of catalyst, these potent organocatalysts contribute to reaction efficiency improvement, perform ROP under atmospheric pressure at room temperature and prevent residual metal contamination from organometallic catalysts, thus showing extraordinary advances and providing a powerful strategy in polymer synthesis. Nevertheless, the resultant polymer is usually with limited molecular weight (10,000–50,000 g·mol^−1^). The acute toxicity and high cost of some organocatalysts (e.g., 1,8-Diazabicyclo[5.4.0]undec-7-ene and 4-Dimethylaminopyridine) are also noteworthy [36]. Therefore, the particularly safe operation process, proper recovery method of organocatalyst system and its economic flexibility in industrial application require further study and environmental assessment.

Besides, a novel and versatile potassium-based catalyst was reported to produce high molecular weight PLA (98,400 g·mol^−1^) in 20 min at room temperature [30]. However, according to the proposed mechanism, the final product probably was not stable due to the double-carbon bonds at the end group [30]. Therefore, this new promising catalyst might need further investigation to confirm. Moreover, enzyme was also employed as a green biocatalyst by which PLA with *M*_w_ 37,800 g·mol^−1^ was harvested without organometallic risk or contamination [35]. Novozyme 435, a typical commercial enzyme in esterification and transesterification, has been applied in biodiesel production. This study originally revealed its catalytic effect in ROP with ionic liquid as low-toxic solvent at moderate reaction temperature (90 °C), providing a viable alternative in catalyst system. Nonetheless, compare to metal catalysts, the low yield (63.2%) and longer process time (7 days) weakened the competitiveness of enzyme catalysts. The employed enzyme amount (10−15 mol %) was much higher than the organometallic catalyst or organocatalysts, so the feasibility of enzyme/ionic liquid system recycle and its activity in reuse deserve more attention to enhance its sustainability and environmental advantage as a green catalyst.

## 5. Newly Developed PLA Modification Techniques

**Figure 3:**
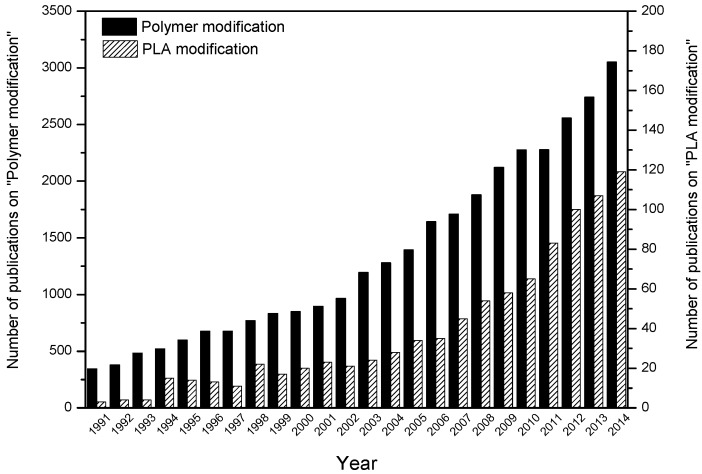
Number of publications with keyterms “Polymer modification” or “PLA modification” in the period of 1991 to 2014.

The natural properties of polymer produced are usually not satisfactory for particular or special purposes in applications. In fact, consumption of PLA always falls behind its production. In 2006, 140 kilo tonnes (KT) PLA was produced by Cargill in USA, while only 60 KT was consumed [17]. This is mainly due to the limited properties of PLA, such as poor thermal stability and hydrophilicity [39], brittleness and high crystallinity [1], preventing its further application as replacement of petroleum-plastics. For instance, less than 10% elongation at break limits PLA material’s application in which plastic deformation at high levels is demanded [40]. To overcome its natural limitations, several modification techniques have been studied to improve the quality of PLA plastic products. Contrary to decreasing attention on PLA synthesis methods, the studies on polymer modification and PLA modification techniques both keep exponential increase in the recent decade as shown in Figure 3.

Generally, modification includes bulk modification and surface modification [41,42], in which the detailed techniques are presented as follows.

### 5.1. Bulk Modification

Bulk modification influences the chemical composition and structure of PLA by copolymerization with other monomers or blending with other polymers [43]. In the existence of carboxyl and hydroxyl groups, lactic acid is available to be copolymerized with other monomers by polycondensation to produce low molecular weight copolymers [41]. Poly(lactic acid-*co*-glycolic acid) (PLGA) was suggested to improve biodegradability with lower melting point and stronger solubility, hence it has been applied as the best defined biomaterial with great performance in drug release application [1]. The degradation characteristic could be adjusted by controlling the ratio of monomers, *i.e.*, lactic acid and glycolic acid [44]. Thermal stability and elastic property could also be reinforced by introducing biomesogenic units [45]. Lactide is also accessible to other cyclic or linear monomers (e.g., glycolide, trimethylene carbonate) in ring opening copolymerization for high molecular weight copolymers. Furthermore, combination of lactide in different stereoisomers (l-lactide, d-lactide and meso-lactide) has significant effect on PLA thermal and mechanical properties [46]. In 2005, Bigg *et al.* observed elongation at break and tensile strength of PLA increased by poly(l-*co*-meso-lactide) at 80%/20% [47].

In addition, the blending of PLA with other bio-polymers [e.g., chitosan, poly(ethylene glycol) (PEG), poly(e-caorolactone) (PCL)] could also contribute to property improvement [5]. For instance, blending of PLA with PCL has been reported with enhanced flexibility but reduced toughness [5]. Poly(meso-lactide-*co*-g-chitosan) was reported to reinforce the adsorption of drugs and adhesion of cells as a new biomedical material [48].

Moreover, controlling the end group of PLA by copolymerizing with diols or anhydride to eliminate hydroxyl or carboxyl groups at the endpoint could improve molecular weight, resulting in better thermal stability [49].

In summary, bulk modification is usually used to improve toughness, stability and degradation rate.

### 5.2. Surface Modification

As PLA’s surface properties are crucial to its application, surface modification could render desirable surface properties. Different from bulk modification, surface modification usually serves to promote hydrophilicity, roughness and to introduce reactive groups [41]. Plasma polymerization, surface coating, entrapment and radiation induction are newly developed methods in this area [43]. There has been numerous research and review about these techniques in recent years, so this review will just focus on some latest cases.

In entrapment method, alginate, chitosan and gelatin have been entrapped on PLA surface in reversible swelling process to associate bio-macromolecules onto PLA by enhancing adsorption [50]. For plasma polymerization, Park *et al.* reinforced ultimate tensile strength of PLA to 2 MPa by chemical modification using oxygen plasma treatment, and successfully made nanofiber scaffold with this modified PLA material in 2007 [51]. In 2014, Jordá *et al.* proved the effectiveness of air plasma treatment in surface energy improvement, by which the surface energy of PLA was increased from 37.1 to 60 mJ·m^−2^, promoting its further adhesion uses [52]. Moreover, biodegradation of PLA and PLGA has been successfully improved further by e-beam radiation to promote their performance in medical device, e.g., orthopedic fixation [53]. In 2014, Norazlina reviewed PLA/graphene-based nanocomposites as reinforcement materials with improved thermal, electrical and mechanical properties, which are suitable for applications in electronic circuits, sensors and electrodes [54]. Chao *et al.* also proposed an improved alkali-acid hydrolysis method, which enhanced hydrophilicity of PLA by using citric acid as the washing solution to increase PLA surface roughness significantly, and to decrease PLA film’s surface water contact angle at the same time [55].

Overall, most of polymer modifications aim at a desired property for specific commodity products. The abundant successful cases indicate that further research in this area is promising to unlock future market of PLA and other biopolymers.

## 6. Comparison and New Trends of Poly(Lactic Acid) Synthesis and Modification Development

In summary, polycondensation is mostly used to produce PLA with low molecular weight using basic equipment and process, while ROP aims for production of a wider range of molecular weight polymer by controlling the purity of lactide and its polymerization. Since each approach has its unique advantages and limitations, the selection of a specific method should be based on the application. For instance, drug release materials favor low molecular weight PLA as it could be degraded quickly. While high molecular weight PLA is suitable to produce packaging and textile products. In the light of current development, the advantages and disadvantages of different methods presented in this review are summarized in Table 4.

Generally, demanding synthesis conditions, such as high temperature heating (150–250 °C) under vacuum pressure for long time, are essential and requiring precise control at this stage. Therefore, based on the discussions presented above on the newly developed synthesis methods of ROP and polycondensation, two crucial factors deserve further investigation. The first factor is high-performance catalyst, such as organocatalyst and potassium-based catalyst mentioned above, could perform polymerization efficiently at room temperature under atmospheric pressure, reducing most of the energy cost required by conventional methods. Furthermore, as compared to single catalyst (e.g., stannous octoate), binary catalysts showed better synergic effect at appropriate condition [57]. On the other hand, it would be more environmental benign if metal-based catalysts could be replaced by green catalysts (e.g., low-toxic organocatalyst and enzymes).

The second factor is equipment with high efficiency heating system, which is usually necessary to obtain PLA with high molecular weight (>100,000 g·mol^−1^). For instance, microwave heating accelerated both PLA polycondensation and lactide synthesis [10,24]. Short path distillation and screw extruder were also suggested as effective setups to demonstrate immediate separation of product from reaction system [19,26].

**Figure 4:**
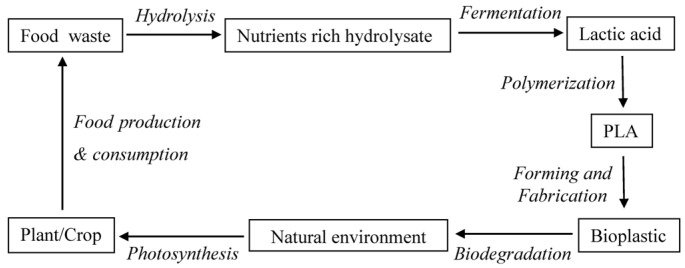
Flow chart of PLA production from food waste.

Apart from the techniques in polymerization and modification, source of lactic acid is another significant factor contributing to PLA production cost. Currently, lactic acid is usually produced by bacterial or yeast fermentation using carbohydrates (e.g., glucose) from agricultural crops such as corn [58]. To reduce the cost of raw material, cheaper alternatives, such as agricultural residues and food waste are suggested [59,60]. In our previous study, food waste has been reported as high nutritional resource for production of chemicals and fuels [61]. Nowadays, research on PLA synthesis using lactic acid derived from food waste (Figure 4) is undergoing in our group. The project is titled as “Conversion of Food Waste into Polylactic acid Fibre”, building for a closed life cycle in which PLA fiber is produced for textile applications using food waste as cost-free feedstock.

In this study, both polycondensation and ROP have been investigated. In order to reduce carbonization and possible side reactions caused by trace impurities from fermentation broth, process with low temperature is desired. For polycondensation, we designed a new heating program consisting of two stages in which different temperatures and inner pressures were served. It has been found that this new method could improve PLA molecular weight (>30,000 g·mol^−1^) efficiently under moderate conditions. In ROP method, nanoparticles of metallic oxide has presented higher catalytic efficient than tin-octoate in lactide synthesis, probably due to more sufficient surface area. The new nanoparticle catalyst is of lower toxicity and less metal contamination as compared to organotin. This study is currently under investigation in our laboratory.

Further to laboratory research, the feasibility of applying newly developed synthesis methods in industry using organic waste as raw material needs detailed evaluation, such as techno-economic analysis and environmental impact assessment [62].

In terms of modification, since most techniques are recently developed, its further investigation requires systematic principles based on PLA and other polymers’ market demands and areas of improvement. Furthermore, the evaluation on economic feasibility of modification techniques at industrial scale is still in progress and is eager for thorough investigation.

## 7. Conclusions

PLA synthesis has been developed over 80 years and has achieved its industrial production. However, its demanding manufacturing operations lead to high cost and limit product properties, which impede its further development and competition with petroleum-derived plastics. At this stage, production techniques in both polycondensation and ring-opening polymerization have been improved a lot by modified synthesis methods. The recently developed methods in the period of 2005–2015 are discussed and summarized in this review for potential enhancement in PLA and other polymers synthesis.

From the authors’ perspective, high performance catalyst and high efficiency heating system are of the most desired elements for polymer synthesis in greener manner. Modification of PLA product by copolymers and nanocomposites presents good potential in a broad variety of applications. Techno-economic evaluation and environmental impact assessment are also necessary to fill the gap between laboratory research and industrial production.

## Figures and Tables

**Table 1:** Newly developed laboratory methods of polycondensation.

Method	Catalyst	Solvent	*T* (°C)	*P* (kPa)	Duration (h)	Yield (%)	*M*_w_ (g·mol^−1^)	Ref.
DP	Nil	Nil	200	1.37	>100	34.52	90,000	[9]
DP	Stannous chloride (0.6 mol %)	TsOH ^a^	200	4	0.5	54	16,000	[10]
AP	Stannous octoate (0.2 wt %)	m-Xylene	138	Normal	72	-	33,000	[11]
SSP	(1) ^c^ Stannous chloride (0.1 wt %)	TSA ^b^	180	-	5	99	36,000	[8]
	(2) ^c^ Nil	Nil	130–160	0.07	30	68	202,000	
SSP	(1) ^c^ Stannous octoate (0.05 mol %)	TSA ^b^	150–180	1.3	10–12	84	46,000	[12]
	(2) ^c^ Nil	Nil	120–200	0.6	10–30	63	102,000	

^a^: *p*-toluenesulfonic acid; ^b^: *p*-toluenesulfonic acid monohydrate; ^c^: (1) conditions in melt-polycondensation stage; (2) conditions in solid state polycondensation stage.

**Table 2:** Newly developed laboratory synthesis methods of lactide.

Catalyst	Set up/Equipment	*T* (°C)	*P* (kPa)	Duration (h)	Yield (%)	Ref.
Acidified chloride catalyst supported on silica gel	Silica gel catalyst system with nitrogen gas flow	170–260	0.08–10	4–12	60	[18]
Stannous octoate (3 wt %)	Short path distillation	170–250	0.5	-	95–97	[19]
Zinc oxide (1 wt %)	Heating gas stream	230–240	1.33–26.6	-	72	[20]
Stannous octoate-toluene (0.04 wt %)	Oil bath with nitrogen gas flow	220–240	11.3	6–8	80	[21]
Zinc oxide-stannous octoate	Constant temperature heating device	180–206	95	7	86.4	[22]
Stannous oxide (0.1 wt %)	Oil bath	220	2.67	>8	77	[23]
Zinc powder (1.2 wt %)	Modified domestic microwave oven	170–180	3.99	3–5	40.3	[24]
Stannous octoate (1 wt %)	Oil bath and ice bath	180–210	1.33	16	41.3	[25]

**Table 3:** New developed methods of lactide polymerization.

Catalyst	Solvent	*T* (°C)	*P* (kPa)	Duration (h)	Yield (%)	*M*_w_ (g·mol^−1^)	Ref.
Stannous octoate (0.04 wt %)	Nil	170	Normal ^a^	2	91–93	172,663	[19]
Stannous octoate	PEG ^b^	180–185	Nitrogen flow	7 min	97–99	93,300	[26]
Stannous octoate (0.05 mol %)	PGL-50 ^c^	160–200	Nitrogen flow	3–5	95–96	468,000	[27]
Aluminum isopropoxide	Nil	130	Nitrogen flow	48	94.8	24,900	[28]
Stannous octoate (0.03 wt %)	1-dodecanol	140	10^−3^	10	>95	100,000	[29]
Potassium hexamethyl-disilazide	Toluene	25	-	20 min	100	98,400	[30]
-	PDP ^d^	180–210	1.33	16	41.3	28,000	[31]
TBD ^e^ (0.1 mol %)	CH_2_Cl_2_, 4-pyrenebutanol	25	-	1 min	95	62,600	[32]
Thiourea amine (5 mol %)	CH_2_Cl_2_	25	-	105	98	42,000	[33]
SIMes ^f^	THF	25	-	10 min	87	16,500	[34]
Novozyme 435 (10 wt %)	Ionic liquids	90	4 × 10^−5^	168	63.2	37,800	[35]

^a^: atmospheric pressure (101 kPa); ^b^: polyethylene glycol; ^c^: polyglycerine-50; ^d^: 4-pyrrolidino-pyridine; ^e^: 1,5,7-Triazabicyclo[4.4.0]dec-5-ene; ^f^: 1,3-Bis(2,4,6-trimethylphenyl)-4,5-dihydroimidazol-2-ylidene.

**Table 4:** Advantages and disadvantages of different polymer synthesis methods.

Method	Advantages	Disadvantages
Azeotropic polycondensation	Low cost	Low yield
	Basic equipment	Low purity (usually with residual solvent and byproducts in polymer)
	Moderate temperature (<180 °C)	Solvent waste and pollution
Solid state polycondensation	High purity (suppression of side reactions)	Low yield
	High molecular weight	Long duration
	Moderate conditions	Complicated operation
Ring opening polymerization	High purity	Low overall yield
	Wide range of molecular weight (2 × 104 to 6.8 × 105 g·mol^−1^) [56]	Long duration
	Availability in high molecular weight	Demanding condition
	Controlled polymer properties	Complicated operation

## Abbreviations

The following abbreviations are used in this manuscript:

AP: Azeotropic polycondensation
BDPs: Biodegradable plastics
DP: Direct polycondensation
*M*_w_: Molecular weight
KT: Kilo tonnes
PCL: Poly(e-caorolactone)
PEG: Poly(ethylene glycol)
PGL: polyglycerine
PLGA: Poly(lactic acid-*co*-glycolic acid)
PLA: Poly(lactic acid)
ROP: Ring-opening polymerization
SIMes: 1,3-Bis(2,4,6-trimethylphenyl)-4,5-dihydroimidazol-2-ylidene
SSP: Solid state polycondensation
*T*: Temperature
TBD: 1,5,7-Triazabicyclo[4.4.0]dec-5-ene
*P*: Pressure
